# Temporal fusion of entangled resource states from a quantum emitter

**DOI:** 10.1038/s41467-025-62130-0

**Published:** 2025-08-15

**Authors:** Yijian Meng, Carlos F. D. Faurby, Ming Lai Chan, Rasmus B. Nielsen, Patrik I. Sund, Zhe Liu, Ying Wang, Nikolai Bart, Andreas D. Wieck, Arne Ludwig, Leonardo Midolo, Anders S. Sørensen, Stefano Paesani, Peter Lodahl

**Affiliations:** 1 Center for Hybrid Quantum Networks (Hy-Q), Niels Bohr Institute, University of Copenhagen, Copenhagen, Denmark; 2 Lehrstuhl für Angewandte Festkörperphysik, Ruhr-Universität Bochum, Bochum, Germany; 3 NNF Quantum Computing Programme, Niels Bohr Institute, University of Copenhagen, Copenhagen, Denmark

**Keywords:** Qubits, Single photons and quantum effects

## Abstract

Fusion-based photonic quantum computing architectures rely on two primitives: i) near-deterministic generation and control of constant-size entangled states and ii) probabilistic entangling measurements (photonic fusion gates) between entangled states. Here, we demonstrate these key functionalities by temporally fusing resource states deterministically generated using a solid-state spin-photon interface. Repetitive operation of the source leads to sequential entanglement generation, whereby curiously entanglement is created between the quantum states of the same spin at two different instances in time. Such temporal multiplexing of photonic entanglement provides a resource-efficient route to scaling many-body entangled systems with photons.

## Abstract (web summary)

Fusion-based quantum computing relies on small entangled resource states that are then fused together probabilistically via linear optical circuits. Here, the authors demonstrate temporal fusion—where resource states generated at different times by the same quantum emitter are fused together—using a spin-photon interface in a quantum dot embedded in a photonic crystal waveguide.

## Introduction

**Fig. 1:**
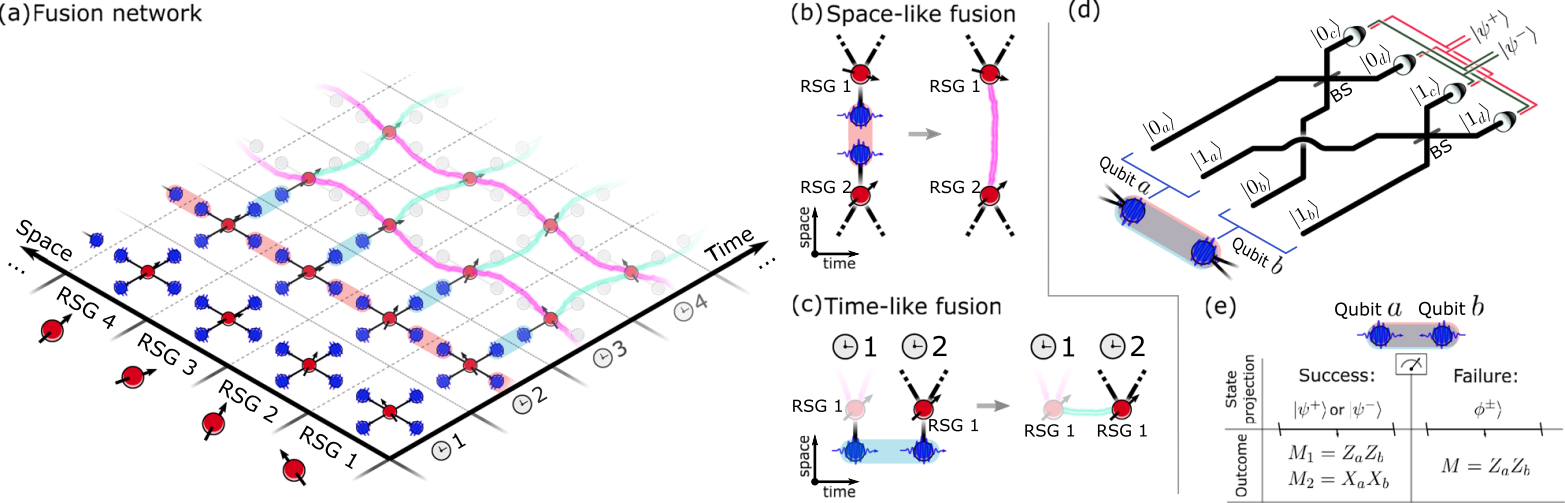
Fusion-based quantum photonic systems. **a** Example of a fusion network generating long-range quantum correlations by sequentially fusing photons from different resource states. ( 1) Resource state generators (RSG), here depicted as quantum emitters, are used to generate constant-size resource states of entangled photons. ( 2) Space-like fusions (red shaded) are used to fuse photons emitted in the same clock cycle (**b**), and ( 3) time-like fusions (blue shaded) fuse photons emitted from the same RSG but at different times using a temporal delay on the earliest photon (**c**). ( 4) The fusion network results in the generation of space-like (purple) and time-like (cyan) quantum correlations. **d** Schematic of a fusion measurement implemented via a probabilistic linear-optical Bell state analyzer with a success probability of 50%. The four two-photon detection patterns corresponding to successful fusion outcomes (*ψ*^±^ projections) are shown, while the remaining two-photon patterns are associated with fusion failure (*ϕ*^±^ projections). **e** Projective states and parity outcomes for a fusion measurement implemented via a probabilistic linear-optical Bell state analyzer for the fusion success and failure cases, with $\left\vert {\psi }^{\pm }\right\rangle=(\left\vert 01\right\rangle \pm \left\vert 10\right\rangle )/\sqrt{2}$ and $(\left\vert {\phi }^{+}\right\rangle \pm \left\vert {\phi }^{-}\right\rangle )/\sqrt{2}=\left\vert 00\right\rangle$ or $\left\vert 11\right\rangle$.

Quantum computing relies on the realization of a universal set of one- and two-qubit gate operations. In photonics, the lack of photon-photon interactions makes deterministic two-qubit gates challenging. This limitation has motivated the development of alternative quantum computing approaches tailored to the photonic platform, where entangling gates can be probabilistically implemented through measurements^1–3^. In this context, fusion-based quantum computing (FBQC) has emerged as a new and resource-efficient approach^4^ where photons are continuously created in small entangled resource states and rapidly measured in shallow linear-optics circuits. Nonetheless, the quantum information survives in the system via quantum teleportation through fusion gates—i.e., entangling two-photon measurements that may be implemented probabilistically with linear-optical circuits^2^. In FBQC, the quantum computing backbone is a fusion network consisting of multiple entangled resource states that are routed from the sources and fused together. Figure 1a illustrates an example of a fusion network of spin-photon entangled resource states^5–8^. The fusion operations either proceed in *space* where two separate resource states are combined (see Fig. 1b) or in time where photons from the same source but emitted at different times are fused (see Fig. 1c). The latter approach applies an optical delay (e.g., in an optical fiber) to interleave subsequently emitted photons, which may offer a significant resource reduction in the required number of physical photon sources^9^. Significant progress has been reported on developing FBQC photonic architectures tailored to hardware capabilities and the physical noises and operations^10–14^.

A central challenge in FBQC is generating the required initial entangled resource states. All-optical approaches use photonic circuits and multiplexing to convert heralded probabilistic linear-optical processes into near-deterministic entanglement generation. However, they require immense hardware overheads that render them highly challenging for near-term technologies^15,16^. Quantum emitters emerge as a platform with a strong potential to surpass these limitations by naturally enabling the deterministic generation of photonic entanglement^17,18^. Quantum emitter platforms, including quantum dots (QDs)^6,8,19–23^, atoms^7,24^, and color centers^25–27^, rely on creating an efficient spin-photon interface where spin-dependent photon generation deterministically entangles the emitted photonic qubits^17^. Recently, the generation of photonic resource states with up to 14 qubits was demonstrated with an ^87^Rb atom trapped in an optical cavity^7^; and subsequently, the authors performed space-like fusions^28^, called cavity-assisted fusion gates, between two individually addressed atoms^29^ to create more exotic states. When implementing FBQC with only space-like fusions, however, the number of individually addressed atoms required scales polynomially with the lattice size. An alternative scheme is the time-like fusion, or temporal fusion, where resource states generated at different times by the same quantum emitter are fused. As such, the number of emitters required drops by a factor of the lattice size (Fig. 1a). This scheme requires delay lines to interleave photons from consecutive resource states. Therefore, emitters with slow photon emission time and long experimental duty cycles^7,29^ necessitate a long delay line, which comes with high optical loss that makes it practically infeasible in these systems.

In contrast to previous works performing fusion in the spatial domain^28,29^, the present work realizes temporal fusion operations, i.e., we fuse consecutive resource states generated from the same QD but at different times. In this process, the state of a single electron spin in the QD becomes entangled with itself, but at two different instances in time. This peculiar entanglement phenomenon is a crucial asset in FBQC since it reduces the required overhead on the number of matter qubits in the architecture^9^ by conveniently using the same QD multiple times. Notably, the computational complexity of time-encoded entangled states is identical to the case of spatial encoding.

## Results

### Experimental scheme

**Fig. 2:**
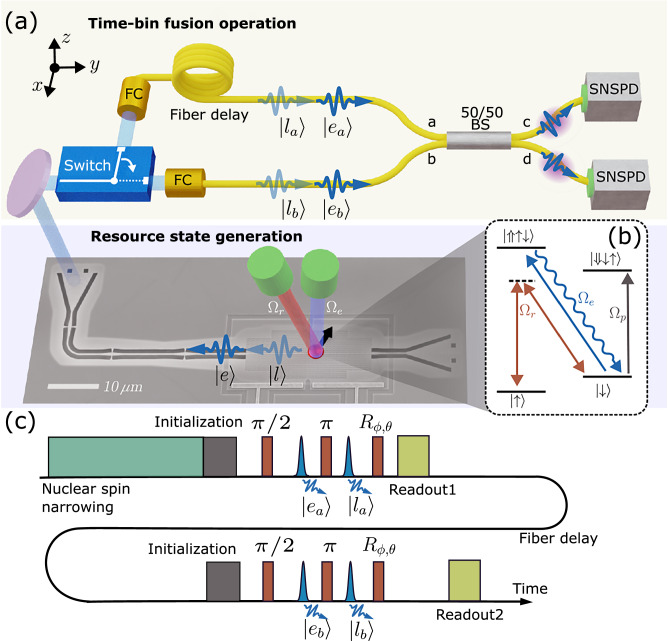
Experimental setup. **a** Schematic diagram of the experiment. The photons generated at time *a* and *b*, 300 ns apart, are entangled with the same quantum dot spin embedded in a photonic crystal waveguide. An electro-optic modulator switches the first photon into path *a* and the second to path *b*. In path *a*, a fiber coupler (FC) collects the photon into a 300 ns fiber delay. The two photons overlap in time at a 50/50 beamsplitter. The joint detection of two photons in path *c* or *d* heralds an entangled state of the spin. **b** Energy level diagram of the quantum dot spin with an electron spin ground state of $\left\vert \uparrow \right\rangle$ or $\left\vert \downarrow \right\rangle$. Controlled Rabi oscillations of the spin state can be achieved through a Raman laser Ω_*r*_. **c** Pulse sequence applied to the quantum dot spin. Before initialization of the spin, nuclear spin narrowing is performed to increase the spin-coherence time. The spin-photon entanglement consists of a *π*/2 and a *π* rotation together with the emission of a photon in different time bins. The spin state can be measured in different bases by rotating the spin before readout.

The experimental setup is outlined in Fig. 2a. It consists of a resource state generator chip, an active switch for routing photons from the emitted resource states, and a fiber-based temporal fusion gate via fiber delay and interference.

The resource state generator is implemented using a spin-photon interface in an InGaAs QD that is embedded in a GaAs photonic crystal waveguide (PCW) (see electron microscope image in Fig. 2a). The QD possesses an optically cycling transition at 947.86 nm and is driven resonantly (Ω_*e*_) for the deterministic generation of single photons (blue excitation in Fig. 2) with a near-unity collection efficiency into the PCW^30^. The QD is deterministically charged with a single electron spin through a bias voltage and we apply an external magnetic field of 4 T along  + *y* direction (Voigt geometry) to access the two Zeeman spin ground states $\vert \downarrow \rangle$ and $\vert \uparrow \rangle$, see Fig. 2b. Coherent Rabi spin rotations are implemented by driving the QD with an off-resonant Raman laser Ω_*r*_ at 650 GHz red-detuning from the optical transition. To improve spin-coherence time, nuclear spin noise is mitigated by implementing nuclear spin narrowing at the beginning of each experimental round^8,31^. The spin is deterministically initialized by a third laser Ω_*p*_, which drives the non-cyclic transition $\vert \downarrow \rangle \leftrightarrow \vert \Downarrow \downarrow \uparrow \rangle$. During the 100 ns duration of the initialization pulse, the emitter is bound to decay to the state $\vert \uparrow \rangle$ through the cycling diagonal transition. Accurate initialization of the spin state is essential for achieving a high entanglement fidelity in our protocol.

The protocol proceeds by generating deterministic spin-photon entanglement^17^. In the present case, the photonic qubit is defined by whether the photon is emitted in an early ($\left\vert e\right\rangle$) or a late ($\vert l \rangle$) time bin. We first apply a spin *π*/2-rotation pulse with spin Rabi frequency Ω_*r*_ to prepare the superposition $\vert \Psi \rangle=(\vert \uparrow \rangle+\vert \downarrow \rangle )/\sqrt{2}$. We then drive the cycling transition $\vert \downarrow \rangle \leftrightarrow \vert \Uparrow \uparrow \downarrow \rangle$ with an optical excitation pulse to emit a photon in the early time bin, followed by a spin *π*-rotation, and then the late excitation pulse. This results in a spin-photon Bell state $\vert {\psi }_{sp}^{-}\rangle=(\vert \uparrow e\rangle - \vert \downarrow l \rangle )\sqrt{2}$ ^8,21^. The spin-echo reshaping *π*-pulse ensures that the protocol is robust towards spin dephasing^32,33^. For each qubit, the early and late time bins are separated by 41 ns. The pulse sequence is shown in Fig. 2c and is repeated after 300 ns to generate two separate resource states for the fusion experiment. Following the nuclear spin narrowing pulse, the spin state is initialized by resonantly exciting the optical transition with a laser pulse (Ω_*p*_), followed by a sequence of alternating spin rotations and optical excitation/emission processes. Subsequently, the spin state is read out by 100 ns of optical pumping of the diagonal transition $\vert \downarrow \rangle \to \vert \Uparrow \uparrow \downarrow \rangle$, see Fig. 2b. This procedure leads to photon emission if the spin is in the state $\vert \downarrow \rangle \equiv \vert 0\rangle$, leading to measuring the spin state in the computational (Pauli *Z*) basis. While the high cyclicity of the diagonal transition enables single-shot readout, the spin readout is probabilistic due to  ≈1% photon collection efficiency. To measure the spin state in other Pauli bases, we use a rotating pulse *R*_*ϕ*,*θ*_ at the end of the resource state generation sequence and prior to the optical spin pumping. The spin is then reinitialized to generate a second resource state and is read out a second time.

The fusion experiment initially generates two separate spin-photon entangled resource states at different instances of time *t*_*a*_ and *t*_*b*_ (*t*_*b*_−*t*_*a*_ = 300 ns), i.e. $\vert {\psi }^{-}\rangle ({t}_{i})=(\vert {1}_{i}\rangle \vert {e}_{i}\rangle - \vert {0}_{i}\rangle \vert {l}_{i}\rangle )/\sqrt{2}$, *i* = *a*, *b*. Here, the qubit state $\vert 0\rangle$ ($\vert 1\rangle$) corresponds to the $\vert \downarrow \rangle$ ($\vert \uparrow \rangle$) spin state, and $\vert e \rangle$ ($\vert l \rangle$) denotes the emitted photon occupying the early (late) time-bin.

Subsequently, the first photon (labeled *a*) and the spin readout signal are routed with an electro-optic switch to a single-mode fiber delay matching the 300 ns time difference between the two resource state generation times. The second photon is routed to a single-mode fiber without implementing a delay, resulting in both time-bin photonic qubits arriving simultaneously at a balanced fiber beamsplitter (BS), as shown in Fig. 2a. Photon detection on the output modes is implemented via superconducting-nanowire single-photon detectors that resolve the early and late photon arrival times. To optimize the signal-to-noise ratio, a pair of etalon filters with a 3 GHz full-width at half maximum is placed before the BS.

In this circuit, the two early components $\vert {e}_{a}\rangle$ and $\vert {e}_{b}\rangle$ of the two time-bin qubits interfere at the BS, and the same is the case for the late components $\vert {l}_{a}\rangle$ and $\vert {l}_{b}\rangle$. By identifying $\vert {e}_{i}\rangle$ ($\vert {l}_{i}\rangle$) with $\vert {0}_{i}\rangle$ ($\vert {1}_{i}\rangle$) as the computational state of each time-bin photonic qubit, the implemented scheme corresponds to a time-bin implementation of the photonic fusion circuit in Fig. 1d and e. Labeling the two output modes of the BS as *c* and *d* (see Fig. 2a), the successful fusion outcomes correspond to measuring the two photons in the detection patterns $\vert {e}_{c}{l}_{d}\rangle$ and $\vert {e}_{d}{l}_{c}\rangle$ for a Bell state projection into *ψ*^−^, and $\vert {e}_{c}{l}_{c}\rangle$ and $\vert {e}_{d}{l}_{d}\rangle$ for projecting into *ψ*^+^. The remaining detection patterns are associated with fusion failure, i.e., projection of the joint state of the two photons into the subspace spanned by the Bell states *ϕ*^±^.

### Verification of temporal photonic fusion

A photonic fusion gate consumes two fused photons from different resource states to generate quantum correlations between the remaining qubits. In our case, these correlations are generated between the two spin qubits encoded in the QD spin at times *t*_*a*_ and *t*_*b*_, as depicted in Fig. 1c, representing a time-like fusion operation. The two resource states are spin-photon Bell states $\vert {\psi }^{-}\rangle ({t}_{i})$ and a successful fusion measurement projects the photons into *ψ*^+^ (*ψ*^−^) to generate the joint two-spin states ${\vert {\psi }^{\pm }\rangle }_{s}=(\vert {\uparrow }_{a}{\downarrow }_{b}\rangle \pm \vert {\downarrow }_{a}{\uparrow }_{b}\rangle )/\sqrt{2}$. This corresponds to a spin entangled with itself at two different instances of time. These states are stabilized by the joint Pauli operators  −*ZZ*,  ±*XX*, and  ±*YY*, i.e., each represents the unique common eigenstate of eigenvalue +1 for these commuting operators^34^. Upon fusion failure (*ϕ*^±^ projection), the *ZZ* fusion outcome is still obtained, but the *XX* and *YY* outcomes are erased. This results in the generation of perfect correlations between the spin qubits only in the *ZZ* basis (with expectation value  +1), but no correlations in the *XX* and *YY* bases.

**Fig. 3:**
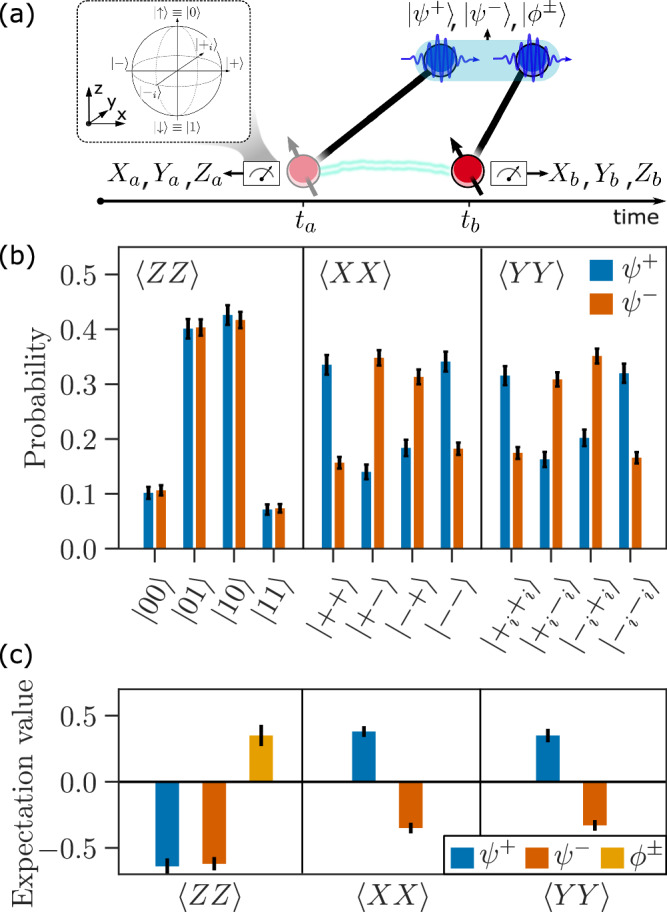
Fusion measurement results. **a** The performance of the time-like fusion is probed by analyzing the entanglement fidelity of the two spin states after fusing the two photons in the resource states. **b** Spin entanglement correlation measurements are shown for the successful fusion outcomes where the photons are projected into *ψ*^+^ (blue) and *ψ*^−^ (red) for the different eigenstates of the spin Pauli basis: $\{\left\vert 0\right\rangle \equiv \left\vert \downarrow \right\rangle,\left\vert 1\right\rangle \equiv \left\vert \uparrow \right\rangle \}$ the eigenbasis of *Z*, $\{\left\vert \pm \right\rangle=(\left\vert 0\right\rangle \pm \left\vert 1\right\rangle )/\sqrt{2}\}$ the eigenbasis of *X*, and $\{\left\vert {\pm }_{i}\right\rangle=(\left\vert 0\right\rangle \pm i\left\vert 1\right\rangle )/\sqrt{2}\}$ the eigenbasis of *Y*. **c** Expectation values of the spin state stabilizers conditioned on success (*ψ*^±^) and failure (*ϕ*^±^) of the photonic fusion. Due to the erasure of the *XX* and *YY* fusion outcomes upon failure, only the *ZZ* value is shown for this case. All error bars are estimated from Poissonian photon statistics.

The correspondence between the fusion outcomes and the resulting joint state of the spin qubits described above enables us to probe the fusion operation by measuring the quantum correlations generated between the spin qubits. Such an analysis is performed by measuring the states of the two spin qubits in different single-qubit Pauli bases to obtain the shared quantum correlations, as depicted in Fig. 3a. The measured correlations between the spin qubits for the *ZZ*, *XX*, and *YY* Pauli operators conditioned on successful fusion outcomes *ψ*^±^ are shown in Fig. 3b. Because these operators are the stabilizers of the targeted final joint state, their measurements enable benchmarking the fusion performance by verifying the presence of entanglement between the early spin qubit and the late spin qubit. In particular, they can be used to calculate the fidelity with the target state using standard analysis techniques^35^. We find ${{\mathcal{F}}}=0.57(1)$ that when conditioning on the fusion outcome *ψ*^+^ and ${{\mathcal{F}}}=0.57(1)$ when conditioning on *ψ*^−^. Both cases are significantly above the 50% bound, indicating genuine spin qubit entanglement.

The ability to perform time-like fusion extends the standard entanglement in space to the time dimension. Interestingly, the possible separation in time between the two spins is limited by any propagation loss in the optical fiber rather than the spin-coherence time. Consequently, the spin-spin entangled states can be long-lived, i.e., the 50% loss propagation distance for a photon at our experimental wavelength of 950 nm corresponds to 7 μs optical delay^36^, whereas at a more optimized telecom wavelength (C-band)^37^, the same loss corresponds to a much longer delay of 90 μs optical delay^38^. In contrast, the spin-coherence time (*T*_2_) of an InGaAs QD spin is typically 2 μs^39^. This can be understood since, in-between the photon generation processes, the coherence between the spins at different times is erased by the spin initialization pulses. However, the photon fusion operations can be seen to recover the spin coherence, and the time-like fusion gate can be interpreted as a heralded quantum memory operation. In this process, the spin state is collapsed and reinitialized between the two time instances, while the quantum information prevails in the photon initially entangled with it, and the fusion operation effectively teleports it into the new spin state. Since this operation is mainly limited by the photon propagation loss, the coherence of quantum information can be kept longer than that of the spin. This ability to interleave quantum information in optical delay lines is a major resource when scaling up photonic quantum processors.

### Benchmarking the performance of fusion operations

An important metric to benchmark the functionality of fusion operations in a network is the noise rate of the fusion outcomes, i.e., the rate with which erroneous results are obtained in the parity checks^4^. Due to the correspondence between the fusion outcome and the joint spin state described above, the error rates in a Pauli operator associated with a fusion outcome can be probed by analyzing the error rates in the same operator but on the spin qubits. Note, however, that estimating fusion error rates through the spin states introduces additional imperfections due to noise (e.g., rotation and readout errors) in the spin system. The obtained error rates should therefore be considered upper bounds on the intrinsic performance of the photonic fusion gate. Expectation values for the *ZZ*, *XX*, and *YY* spin operators conditioned on the successful fusion outcomes *ψ*^±^ are reported in Fig. 3c. For the *ψ*^+^ fusions, the corresponding error rates are 17(2)%, 32(2)%, and 36(2)% for *ZZ*, *XX*, and *YY*, respectively, and conditioning on *ψ*^−^ leads to 18(2)%, 34(1)%, 34(1)%. In the same figure, we also report the *ZZ* expectation value conditioned on the fusion failure outcome *ϕ*^±^, obtained from detection patterns with both photons detected in either the early or the late time bin. The *XX* and *YY* operators are erased in this failure case and thus not reported. These events have a higher contribution from residual background photons (see Supplementary Information), resulting in a higher *ZZ* error rate of 34(3)%. Supplementary Information, we report an analysis of physical mechanisms that contribute to the error rates and show that a large portion of the noise budget is expected to arise from spin noise. Routes to further improvement of the experimental performance have been discussed in detail in ref. ^8^.

## Discussion

We have demonstrated temporal fusion between two spin-photon Bell states generated by a deterministic quantum emitter. The current physical platform possesses many promising attributes towards the implementation of FBQC, namely a fast photon generation rate, high photon indistinguishability, and active spin control. The short photon radiative lifetime (250 ps) and control pulse sequence enable the implementation of temporal fusions using a short optical delay with low propagation loss, a major advantage over atomic platforms with slower lifetimes (~μs). Importantly, although the current implementation consists of only four qubits, our physical platform is fully compatible with generating resource states of more photonic qubits, which, when combined with fusion gates, would allow percolation of entanglement across the fusion network. In particular, active spin control provides the reconfigurability of spin control pulse sequence necessary for the generation of different classes of multi-photon resource states, including GHZ states^8^, linear cluster states, star graphs, and redundantly encoded cluster states. Our results, therefore, demonstrate the key components towards realizing FBQC with lower overhead.

Although the current experiment demonstrates the coincidence measurement of the highest number of qubits using a QD emitter to date, a viable resource state for fault-tolerant measurement-based quantum computation will likely require at least four qubits^40^. In this context, achieving fusion and direct fidelity measurement with FBQC-viable resource states would necessitate coincidence measurements involving more than 7 qubits, highlighting the need for significant improvements in resource state generation and collection efficiency.

Our proof-of-concept demonstration may be further improved by advancing the spin system and, in particular, strain-free GaAs droplet-epitaxy QDs^41,42^, with reduced high-frequency nuclear spin noise, appears an attractive route to significantly improve the system performance. Additionally, heterogeneous integration with lithium niobate or silicon-based chips would enable active elements with low-loss optical delay^43,44^, further boosting collection efficiency and paving the way for scalability to support the generation of larger source states^45^.

The thresholds for photon loss, photon distinguishability between emitters, and spin noises have been recently simulated based on our current entanglement source^46^. While the thresholds for fault-tolerant computation are out of reach for the current system, there is a clear path for further development of both quantum photonic hardware and fusion-based architectures based on deterministic quantum emitters^8^.

Furthermore, the optimization of tailored architectures that can take advantage of the spin-photon building block for quantum photonic hardware may bring hardware requirements closer to near-term technology^47,48^. A main advantage of the photonic approach is that large entangled states can be built from a few hardware components (tens to hundreds of quantum emitters^9^) by repetitions of just two primitives: near-deterministic resource state generation and fusion operations. We have reported the first experimental demonstration of both functionalities, constituting an important step towards scalable fusion-based photonic quantum technologies.

## Supplementary information

Supplementary Information

Transparent Peer Review file

## Data Availability

The data underlying the results presented in this paper are available at the link.
