# Supplementary material for: Temporal fusion of entangled resource states from a quantum emitter
Source: Nat Commun. 2025 Aug 15;16:7602. doi: 10.1038/s41467-025-62130-0 (PMC12356959; doi:10.1038/s41467-025-62130-0)
Supplement: Supplementary file 1 — Supplementary Information [file 41467_2025_62130_MOESM1_ESM.pdf]

Supplementary Materials - Temporal fusion of entangled resource states from a quantum emitter

Yijian Meng,[1] Carlos F.D. Faurby,[1] Ming Lai Chan,[1] Rasmus B. Nielsen,[1] Patrik I. Sund,[1] Zhe Liu,[1] Ying Wang,[1] Nikolai Bart,[2] Andreas D. Wieck,[2] Arne Ludwig,[2] Leonardo Midolo,[1] Anders S. Sørensen,[1] Stefano Paesani,[1,3,*] and Peter Lodahl[1,†]

[1] *Center for Hybrid Quantum Networks (Hy-Q), Niels Bohr Institute, University of Copenhagen, Blegdamsvej 17, Copenhagen 2100, Denmark*

[2] *Lehrstuhl für Angewandte Festkörperphysik, Ruhr-Universität Bochum, Universitätsstrasse 150, D-44780 Bochum, Germany*

[3] *NNF Quantum Computing Programme, Niels Bohr Institute, University of Copenhagen, Blegdamsvej 17, Copenhagen 2100, Denmark.*

EXPERIMENTAL SETUP

In Fig.1, we show an extended illustration of the experimental setup used to collect and interfere single photons from the quantum dot sample. A pair of etalon filters (3 GHZ full-width half maximum) is employed to maximize the signal-to-noise ratio, i.e, filter out photon emissions from unwanted optical transitions. After transmission through the etalon filters, a half-wave plate (HWP) and a quarter-wave plate (QWP) are used to maximize reflection at the first polarization beamsplitter (PBS). A combination of an electro-optic modulator (EOM, Eksma Optics pulse picker UP1), a HWP, and a PBS acts as an optical switch, i.e, an early photon a goes through an additional 300 ns fiber delay and interferes with a late photon b deterministically. A successful fusion event is heralded by a joint detection of two photons on the two output ports c and d of a 50/50 non-polarizing beam splitter (BS). We employ a time tagger (Swabian instrument, Time Tagger Ultra) to record photon detection events registered by the superconducting nanowire single-photon detectors (SNSPD).

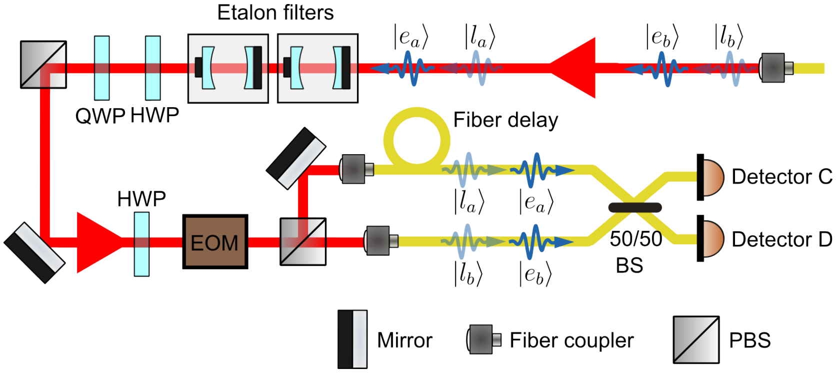

Supplementary Figure 1. Illustration of the photon collection and state characterization setup.

The quantum dot device for resource generation has been detailed in our previous work [S1]. The device comprises a singular layer of self-assembled InAs quantum dots enveloped by GaAs and embedded within a *p*-*i*-*n* diode in a photonic crystal waveguide. A forward DC bias voltage is applied across the diode to serve the dual purpose of stabilizing the charge environment and deterministically charging the quantum dot with an electron.

* stefano.paesani@nbi.ku.dk
† lodahl@nbi.ku.dk

TIME-BIN ENCODED PHOTONIC FUSION

A fusion operation is heralded by the joint detection of two photons at the detectors. Before the 50/50 fiber beamsplitter (BS), the joint state can be written as the tensor product of two resource states a and b:

$$|\psi_{ab}\rangle = \frac{1}{2}(|1_a\rangle|e_a\rangle - |0_a\rangle|l_a\rangle) \otimes (|1_b\rangle|e_b\rangle - |0_b\rangle|l_b\rangle)\,, \tag{1}$$

where a and b are also used to denote the two input paths of the BS. The photons of the resource state a (b) are directed to the input path a (b) of the BS (see Fig. 2 of the main text).

After the 50/50 BS, the state evolves into:

$$|\psi_{cd}\rangle = \frac{1}{4}(|1_a\rangle(|e_a^c\rangle + i|e_a^d\rangle) - |0_a\rangle(|l_a^c\rangle + i|l_a^d\rangle)) \otimes (|1_b\rangle(i|e_b^c\rangle + |e_b^d\rangle) - |0_b\rangle(i|l_b^c\rangle + |l_b^d\rangle)), \tag{2}$$

where the superscripts c and d correspond to the two output paths of the BS. The state can be written as

$$\begin{aligned} |\psi_{cd}\rangle = \frac{1}{4}\Big[& |1_a 1_b\rangle(i|e_a^c e_b^c\rangle + |e_a^c e_b^d\rangle - |e_a^d e_b^c\rangle + i|e_a^d e_b^d\rangle) \\ & -|1_a 0_b\rangle(i|e_a^c l_b^c\rangle + |e_a^c l_b^d\rangle - |e_a^d l_b^c\rangle + i|e_a^d l_b^d\rangle) \\ & -|0_a 1_b\rangle(i|l_a^c e_b^c\rangle + |l_a^c e_b^d\rangle - |l_a^d e_b^c\rangle + i|l_a^d e_b^d\rangle) \\ & +|0_a 0_b\rangle(i|l_a^c l_b^c\rangle + |l_a^c l_b^d\rangle - |l_a^d l_b^c\rangle + i|l_a^d l_b^d\rangle)\Big]\,. \end{aligned} \tag{3}$$

Detecting the two photons in different time-bins heralds the spin-spin state: $\psi_\pm = \frac{1}{\sqrt{2}}(|0_a 1_b\rangle \pm |1_a 0_b\rangle)$:

$$\langle e^c l^d|\psi_{cd}\rangle = \langle e^d l^c|\psi_{cd}\rangle = |\psi_-\rangle \tag{4}$$

$$\langle e^c l^c|\psi_{cd}\rangle = \langle e^d l^d|\psi_{cd}\rangle = i|\psi_+\rangle. \tag{5}$$

assuming that photons in the two resource states a and b are indistinguishable. The probability of heralding ψ_- or ψ_+ is 25%, giving a total fusion success probability of 50%. An important note is that events where indistinguishable photons arrive at the same time-bin but at different detectors are prohibited by the Hong-Ou-Mandel effect. Imperfect indistinguishability would give rise to these events, which projects the state into a mixed state of $\rho(\phi_\pm) = (|0_a 0_b\rangle\langle 0_a 0_b| + |1_a 1_b\rangle\langle 1_a 1_b|)/2$.

To characterize the fusion operation, the expectation values in different measurement bases are calculated as:

$$\langle ZZ\rangle = \frac{(N_{0,0} + N_{1,1} - N_{1,0} - N_{0,1})}{(N_{0,0} + N_{1,1} + N_{1,0} + N_{0,1})}, \tag{6}$$

$$\langle XX\rangle = \frac{(N_{+,+} + N_{-,-} - N_{+,-} - N_{-,+})}{(N_{+,+} + N_{-,-} + N_{+,-} + N_{-,+})}, \tag{7}$$

$$\langle YY\rangle = \frac{(N_{+i,+i} + N_{-i,-i} - N_{+i,-i} - N_{-i,+i})}{(N_{+i,+i} + N_{-i,-i} + N_{+i,-i} + N_{-i,+i})}, \tag{8}$$

where $N_{m,n}$ are coincidence counts corresponding to the population measured in the spin $|m_a\rangle|n_b\rangle$ basis.

For spin measurement, an additional rotation pulse is employed before the readout pulse to project the spin from an arbitrary state to $|0\rangle$ ($|\downarrow\rangle$), therefore four measurement configurations are required for each basis, see e.g. Eq. (6), where each measurement corresponds to a term in the numerator.

MONTE-CARLO FIDELITY SIMULATIONS

To analyze the impact of experimental imperfections on the state infidelity, we conducted Monte-Carlo (MC) simulations, incorporating error parameters detailed in Supplementary Table 1. The details of MC simulation, including how error sources are incorporated, are described in Ref. [S1]. The outcomes of these simulations for the current experiment are summarized in Supplementary Table 2. To discern the contribution of individual error sources, we initially ran simulations considering all errors, resulting in an entanglement fidelity $\mathcal{F}_o$. We found $\mathcal{F}_o = 52\%$ for both ψ_+ and ψ_- states, which is similar to the experimental results.

The simulation follows the experimental generation and detection procedure, employing 12 measurement configurations to project early and late spin states into one of the three bases: X, Y, or Z. For each spin state, we simulate over 5000 realizations.

To simulate the detection procedure based on two-photon events on the detector, we also include an additional drop in photon visibility attributed mostly to imperfect path delay and polarization drift in the detection interferometer. To evaluate this effect, we perform a Hong–Ou–Mandel measurement[S2], measuring the visibility between early and late photons that are 300 ns apart, resulting in $V_f = 68(1)\%$. We compare this with the visibility measured when the photons are 12 ns apart while other settings are similar, i.e., $V_i = 84(1)\%$[S1]. The relative drop in visibility due to the delay is quantified by the formula $1 - V_i/V_f = 19\%$.

Subsequently, we conducted a second simulation to find the infidelity caused by the different error sources. We denote the lack of a specific error source p, to yield a fidelity $\mathcal{F}_p$. The infidelity attributed to an error source p can be estimated as $\mathcal{F}_p - \mathcal{F}_o$. While this method assumes the errors are independent, it provides a valuable estimate for prioritizing further optimization efforts. Supplementary Table 2 lists the contribution from all known error sources.

The highest contribution comes from off-resonant excitation, which happens when we excite the opposite diagonal transition than the one we are interested in. The two diagonal transitions are separated by 9 GHz, while our pulsed laser used for excitation has a bandwidth of 20 GHz. In order to avoid exciting both transitions we tune the frequency of the excitation laser slightly away from the other transition, but unwated excitations are unavoidable. The next two significant error sources are nuclear spin noise and incoherent spin-flip during rotation pulse.

It should be noted that many of the listed error sources when combined will correlate destructively such that the total error provided by them is more than the sum of their individual errors. For example the negative effect of off-resonant excitation would be magnified by a population increase in the off-resonant spin state under an imperfect spin π-rotation pulse.

**Parameters**	
Radiative lifetime $1/\Gamma$	235 ps
Cyclicity C	36
Spin rotation Q-factor Q	34
Spin readout fidelity F_{r}	99%
Spin initialization fidelity F_{int}	99%
Pure dephasing rate γ_d	0.069 ns^{-1}
Excitation laser detuning	−2 GHz
Frequency splitting between cycling transitions Δ	10 GHz
Excitation pulse shape	Gaussian
Excitation pulse area	0.7π
Excitation pulse duration (FWHM intensity)	30 ps
High-frequency nuclear spin noise	Modelled as in [S1]
Visibility reduction from 300 ns delay	−19%

Supplementary Table 1. List of parameters used for the simulation. The excitation laser detuning is the detuning of the excitation laser relative to the targeted cycling transition. The amplitude of nuclear spin noise is extracted from the spin echo visibility measurement, see Ref. [S1].

[S1] Y. Meng, M. L. Chan, R. B. Nielsen, M. H. Appel, Z. Liu, Y. Wang, N. Bart, A. D. Wieck, A. Ludwig, L. Midolo, *et al.*, Deterministic photon source of genuine three-qubit entanglement, Nature Communications **15**, 7774 (2024).

**All error sources**	**Infidelity**
Off-resonant excitation	8.4 %
Nuclear spin noise	4.8 %
Spin-flip error during rotation	3.9 %
Spin readout error	1.6 %
Visibility reduction from 300 ns delay	1.3 %
Phonon-induced pure dephasing	0.6 %
Off-resonant excitation + Nuclear spin noise	16.6 %
Off-resonant excitation + Spin-flip error during rotation	13.2 %

Supplementary Table 2. Infidelity contribution of all simulated errors. The infidelity contribution due to an error p is calculated as the difference in simulated fidelity with and without the error source p. The last two rows indicate the simulated infidelity when accounting for two error sources simultaneously. The large deviation between combined infidelities and sum of individual infidelities indicates that the error sources are not independent.

[S2] M. H. Appel, A. Tiranov, S. Pabst, M. L. Chan, C. Starup, Y. Wang, L. Midolo, K. Tiurev, S. Scholz, A. D. Wieck, A. Ludwig, A. S. Sørensen, and P. Lodahl, Entangling a hole spin with a time-bin photon: A waveguide approach for quantum dot sources of multiphoton entanglement, Phys. Rev. Lett. **128**, 233602 (2022).
